# Evaluation of Malnutrition and Quality of Life in Patients Treated for Oral and Oropharyngeal Cancer

**DOI:** 10.1155/2021/9936715

**Published:** 2021-08-02

**Authors:** Shruthi Pingili, Junaid Ahmed, Nanditha Sujir, Nandita Shenoy, Ravikiran Ongole

**Affiliations:** ^1^Department of Oral Medicine and Radiology, Sibar Insitute of Dental Sciences, Takkelapadu, Guntur 522509, India; ^2^Department of Oral Medicine and Radiology, Manipal College of Dental Sciences, Mangalore, Manipal Academy of Higher Education, Manipal 576104, Karnataka, India

## Abstract

**Background:**

Oral and oropharyngeal cancer is a debilitating disease with high morbidity and mortality. Depending on the site and extent of the involvement of the cancer and the type of treatment modality, these patients can develop pain, trismus, xerostomia, dysphagia, and taste disturbances, compromising them socially and nutritionally. The aim of the study was to evaluate malnutrition and quality of life in patients treated for oral and oropharyngeal cancer. *Methodology*. A cross-sectional study was conducted which included 97 patients treated for oral and oropharyngeal cancer. The quality of life of the selected patients was assessed by using a validated European Organization for the Research and Treatment of Cancer's Quality of Life Questionnaire, Head and Neck and Mandibular Function Impairment Questionnaire. Pre- and posttreatment weight of the patients were assessed, and weight loss of ≥10% of pretreatment weight was considered as malnutrition. The chi-square test was used to correlate the symptoms with the quality of life. A paired *t* test was used to assess the differences in weight before and after treatment, and a *p* value of <0.005 was considered as significant.

**Results:**

The most commonly reported symptoms were xerostomia (93.81%), pain (81.44%), and dysphagia (76.3%). A total of 40.2% of the individuals in the study had malnutrition. Malnutrition was comparatively lower in the group who had nutritional supplements.

**Conclusion:**

The quality of life in patients treated for oral and oropharyngeal cancer deteriorates immediately after the treatment; however, it significantly improves over time.

## 1. Introduction

In a developing country like India, head and neck cancer is quite common and is ranked as the tenth most common cancer by the International Agency for Research on Cancer [1]. In 2018, global incidence and mortality related to oral and oropharyngeal cancer were estimated to be 447,751 and 228,389, respectively, and in India, incidence and mortality rates were recorded to be 137,895 and 87,569, respectively [2]. The number of oral and oropharyngeal cancer survivors has increased in the recent decade owing to superior diagnostic techniques and advanced treatment modalities [3–5]. Although the number of deaths has decreased, the treatment of oral and oropharyngeal cancer leaves patients compromised physically and mentally [3, 6]. Various treatment modalities of oral and oropharyngeal cancer include surgery, chemotherapy, radiotherapy (RT), or a combination of them [7, 8]. Depending on the site and extent of the involvement of cancer and the type of treatment modality, these patients develop pain, trismus, xerostomia, dysphagia, and taste disturbances compromising them socially and nutritionally. The present study was undertaken with an aim to assess malnutrition and the quality of life (QOL) in patients treated for oral and oropharyngeal cancer.

## 2. Materials and Methods

Sample size calculation: Based on the article by Kamstra et al., [9], the correlation coefficient derived/reported is 0.67. With an alpha error of 0.1% and a power of 99.9%, the *Z* values of the given alpha and beta values are 3.29 and 4.26. With the correlation coefficient and using the abovementioned formula, the required sample size was 87 in number. A prospective study was conducted on 97 patients treated for oral and oropharyngeal cancer after obtaining clearance from the Institutional Ethical Committee (Ref Protocol No. 15121). Inclusion and exclusion criteria for the study are given in Table 1.

Demographic data, pretreatment weight, details including the site, extent, and staging of the cancer, and treatment details including the mode of treatment and duration were retrieved from hospital records. The study population was divided into two groups, namely, the 3 months group and 6 months group, i.e., individuals who came before 3 months and at 6 months for follow-up after treatment. On the day of the study, informed consent was obtained from the patients and they were then clinically evaluated. As part of the study, body weight was assessed and the patient's oral cavity was thoroughly examined to evaluate their mouth opening, dentition, oral hygiene, and for the presence of any mucosal abnormalities. Patients were then asked about the symptoms related to their treatment and were asked to grade their symptoms by using the European Organization for the Research and Treatment of Cancer's Quality of Life Questionnaire and the Head and Neck35 (EORTC QOL-H&N35) and Mandibular Function Impairment Questionnaire (MFIQ). These questionnaires were modified in few areas to suit the Indian population and validated by our resident dietician and oncologist. The quality of life was assessed, and changes in the body weight were correlated with the scores of the questionnaire. All data were coded and transferred to an Excel spreadsheet (Microsoft Corp., Redmond, WA, USA), and a descriptive statistical analysis was performed using SPSS version 20.0 (SPSS Inc., Chicago, IL, USA) with a confidence limit of 80%. Items from both the questionnaires were grouped according to symptoms such as swallowing problems, chewing disabilities, dry mouth, sensory impairment, impaired social activities, and psychological problems. The range of score for each symptom was calculated by adding all the lower and upper limits of questions in that group from both the questionnaires. The range of score obtained for each symptom was divided into 3 groups, namely, mild, moderate, and severe, by extracting an average of the scores of the 2 questionnaires, and the patients with moderate and severe scores were considered to have a significant problem. An example for the averaging of scores is as follows: To address the chewing problem, we have question numbers 4, 5, 6, 10, 12, 13, 14, and 15 from the validated MFIQ and question number 15 from the validated EORTC. Hence, for chewing problems, the minimum and maximum score obtained were 9 and 36, respectively. This range of score is divided into three categories: 9 ≤ 18 which denotes patients having mild problems, 19 ≤ 27 indicating moderate problems, and 28 ≤ 36 denoting severe problems. The categorization of the questions addressing each problem is given in Table 2. The scores and grading for each symptom are given in the Table 3. The chi-square test was used to correlate the symptoms with the quality of life. The paired *t* test was used to assess the differences in weight before and after treatment, and a *p* value of <0.005 was considered as significant.

## 3. Results

Among the 97 patients treated for oral and oropharyngeal cancer, 74.2% of the patients were male and 25.8% were females. The mean age of the study population was 55 (SD ± 10.6) years. 78% of the patients had consumed tobacco either in the smoke or smokeless form, and 60% had consumed alcohol on a regular basis. The site of involvement, staging, and treatment details are summarized in Table 4.

**Figure 1:**
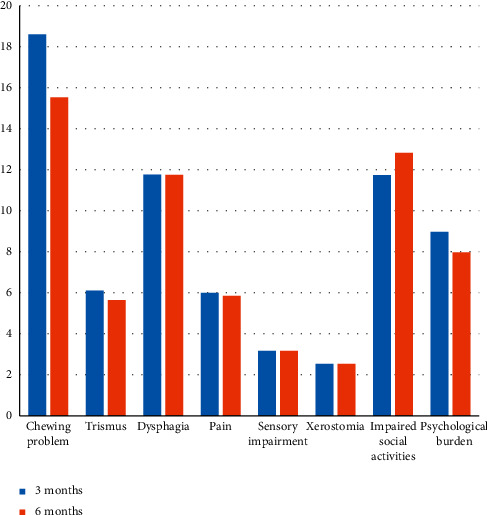
Representing comparison of scores between the 3 months and 6 months group.

The most commonly reported symptoms were xerostomia (93.81%), pain (81.44%), and dysphagia (76.3%). Chewing problems and psychological scores had significantly reduced in the 6 months group when compared to the 3 months group. The comparison of scores for quality of life in the 3 months and 6 months group is shown in Figure 1.

Comparison of weight changes between the two groups showed statistically nonsignificant results with a *t* value of 1.73 but was statistically nonsignificant with a *p* value of 0.098. Malnutrition between the 2 groups was compared, and it was noted that the prevalence of malnutrition was significantly higher (56.4%) in the 3 months group (*p* value 0.04). Only sensory difficulty for taste sensation was significantly associated with malnutrition (*p* value 0.029). The results for association between other symptoms with malnutrition are shown in Table 5.

The results from the chi-square test showed that malnutrition was significantly lower in the group which had nutritional supplements (*p* value 0.033). Chewing problems, dysphagia, and dry mouth (sticky saliva) were significantly observed to be higher among individuals treated with surgery alone, and trismus was significantly higher in patients who were treated with a combination of surgery and RT. The association between the oral symptoms, psychological burden, and impaired social activities with various modes of treatment is given in Table 6.

## 4. Discussion

Cancers affecting the oral and oropharyngeal region and their treatment modalities adversely affect the patients' emotional, physical, and functional well-being. These experiences can deeply scar the patients' lives leading to a dramatic decrease in their QOL and can indirectly cause malnutrition. The QOL assessment is an important tool measuring the outcomes of cancer treatment and has been evaluated in this study.

In our study population, the male-to-female ratio was 2.88 : 1 which is similar to the results of Nagy et al. [1] (2 : 1), Gritz et al. [10] (2.5 : 1), and Hassanein et al. [11] (2.3 : 1). The mean age of the study group was 55 years which is consistent with the results of Nagy et al. [1] (53.8 years), Gritz et al. [10] (58.4 years), and Hassanein et al. [11] (58 years). The most common site of tumor in our study was the tongue (35%) followed by the floor of the mouth (18.6%), oropharynx (12.4%), maxilla (12.4%), and buccal mucosa (10.3%). Our results were similar to studies conducted by Rinkel et al. [12] who stated that the common sites were the tongue (38%), followed by the floor of the mouth (10%).

The most common treatment modality in the present study was the combination of surgery with RT, followed by the combination of surgery, RT, and chemotherapy (CT). This can be explained by the fact that most of the cancers in our study were in stage II and stage III which usually required combined treatment modalities. Combination of surgery with RT was the common mode of treatment in the studies conducted by Rinkel et al. [12] (50%), Kamstara et al. [9] (51.25%), and Nazar et al. [13] (47.2%). In the study conducted by Scharloo et al. [14], the results showed that RT was the commonly employed treatment modality and accounted for 40.7% of the cases. Thomas et al. [15] found that 88.3% of the patients had undergone primary or adjuvant RT. In the investigation carried out by Vartanian et al. [16] on 301 patients, it was found that 52.5% of the patients were treated only by surgery, 11.3% had undergone only RT, and 32.6% were treated with a combination of surgery and RT.

In the present study, the most commonly reported symptoms were xerostomia (93.81%) followed by pain (81.44%) and dysphagia (76.28%). These findings can be explained by the fact that most of the participants were treated by RT. Xerostomia chiefly occurs due to the severe damage and fibrosis of salivary glands caused by RT. These findings were similar to the results of the study conducted by Kamstara et al. [9] who observed that xerostomia was the most common symptom among patients treated for oral and oropharyngeal carcinoma and the other most commonly reported symptoms were trismus and dysphagia. Rathod et al. [17] in their study noticed that xerostomia was the most common symptom associated with the treatment of HNC. The study also revealed that dysphagia and altered taste sensations in oral cancer patients worsened after treatment. A study conducted by De Graeff et al. [18] revealed that the common symptoms associated with treatment of oral and oropharyngeal cancer were pain, difficulty in eating and speech, and sensory impairment. Pain was the second most commonly reported symptom in our study (81.44%). Most of the studies in the literature have suggested pain as the worst symptom experienced as a consequence of cancer therapy [19]. The experience of pain after surgery is described as a nociceptive pain, lasting for a couple of months with moderate improvement over time [20, 21]. Surgical management can also cause tissue and nerve damage resulting in chronic pain syndromes. Terrel et al. [22] in their study among patients treated by mandibular bone resection found that hyperalgesia and allodynia was experienced by approximately 50% and 90% of the patients, respectively. Pain can also be because of the mucositis since most of the patients had undergone the combination of surgery and RT. The systematic review by Trotti et al. [23] stated that the incidence of oral mucositis in patients treated with RT and chemoradiation was very high accounting for 80% of the patients.

In the present study, the scores of various symptoms were lower in the 6 months group when compared to scores of individuals in the 3 months group. These findings suggested that the severity of the symptoms decreased as the duration of treatment increased. The scores of chewing disabilities, trismus, dysphagia, pain, taste disturbances, and psychological scores were comparatively lower among individuals who came for follow-up at 6 month interval. However, only the scores of chewing disabilities and psychological burden were significantly reduced in the 6 months group when compared with the 3 months group. This can be attributed to the prophylactic extraction of teeth and the consequent inadequate healing of the surgical site as a part of the treatment protocol or even a lack of proper prosthetic wear at 3 months interval during treatment follow-up. The improvement at 6 months may be due to the healing of the surgical site in such cases. Adverse effects of treatment such as extraoral surgical scars, pigmented skin, and alopecia which can significantly impact the psychological well-being of individuals are severe immediately after treatment but improve subsequently over time. These treatments also contributed to inability in movement of the tongue resulting in difficulty of speech. All these factors inhibit them from routine activities such as social speaking and social eating and eventually affect them psychologically in due course of the treatment. The scores for sensory difficulties, xerostomia, and impaired social activities are higher in 6 months group when compared with the 3 months group; however, the difference was not statistically significant.

Biazevic et al. [24] recorded the immediate effects of tumor resection in oral and oropharyngeal cancer on the health-related QOL and found reduction in overall rating indicating the improvement of the QOL after 6 months of treatment. It was observed that the commonly impaired functions were chewing difficulties, taste disturbances, pain, and problems associated with swallowing and speech. In the current study, comparison of the scores between 3 months and 6 months showed significantly high scores in chewing problems and psychological burden in the 3-month interval group. Jaw movements for opening chewing and swallowing are correlated with mobility of the tongue and the mandible. Most of our patients were treated for cancer of the tongue and floor of the mouth which have significant effects on chewing and swallowing functions. These are also significantly impaired immediately after the treatment, but improve after a duration of 6 months, which explains the reduction of their scores.

Shepherd et al. [25] conducted a study on oral cancer patients with an aim to examine the impact of surgical, RT, and combination treatment on QOL. In this study, it was found that function reduced immediately after treatment and most functions improved to near baseline levels by 3 months after treatment. Rathod et al. [17] evaluated the outcomes of treatment in HNC patients and noticed that there was a substantial deterioration in the QOL (trismus, xerostomia, pain, and senses) scores 3 months after treatment although the improvement was noted in all the scores by 6 months' time. These findings were very similar to the findings of our present study. Agarwal et al. [26] in a prospective study evaluated changes in the QOL 6 months after the surgical treatment of carcinoma of the tongue and found that there was a significant decrease in scores of the appearance of the patient, dysphagia, chewing, speech, taste, and xerostomia indicating an improvement of the QOL.

Prevalence of malnutrition (weight loss of ≥10%) in our study was 40. 2%, among which 56.4% were in the 3 months group and 43.6% in the 6 months group. Taste disturbances were significantly associated with malnutrition. Altered taste sensation is very distressing for the patients and is associated with decreased appetite and confusion between bitter and sour taste and the inability to discriminate among various tastes. The results of our study were similar to a few other studies in the past. McLaughlin [27] had found that dysgeusia has shown a statistically significant association with malnutrition. Suzuki et al. [28] had revealed that appetite is frequently affected by altered taste sensation along with xerostomia and oral mucositis. Ogama and Suzuki, [29] concluded that patients who were exposed to a cumulative dose of 50 Gy had taste disturbances which severely affected their appetite. However, studies conducted by Jager-Wittenaar et al. [30] and Gellrich et al. [31] showed that dysphagia and chewing problems were mainly associated with malnutrition. Kubrak et al. [32] has suggested that malnutrition may be due to dysphagia and mouth sores caused by the treatment of oral and oropharyngeal cancer.

Our research revealed that malnutrition was absent in a significant number of patients (72.5%) who were on nutritional supplements. Ravasco et al. [33] in a prospective study on HNC patients stated that weight loss is more prevalent in patients who were not receiving dietary counselling when compared to patients who received dietary counselling. van den Berg et al. [34] stated that nutritional interventions during treatment had a positive influence on the outcomes of treatment and resulted in considerably lower malnutrition status in patients treated for oral and oropharyngeal cancer.

## 5. Conclusions

In the present prospective study, we observed that the QOL in patients treated for oral and oropharyngeal cancer deteriorates immediately after treatment but significantly improves over time. Our study also highlights the importance of nutritional supplements, their positive influence on the outcomes of treatment, and their beneficial effects on patients with malnutrition.

## Figures and Tables

**Table 1:** Inclusion and exclusion criteria of the study.

Inclusion criteria
(i) Patients aged above 18 years who were treated for oral and oropharyngeal cancer

Exclusion criteria
(i) Patients with metastatic disease, i.e., stage IV C (ii) Patients treated with cancer below the level of the hypopharynx (iii) Patients with poor performance state, not fit to receive any radical treatment (iv) Patients not fit for concurrent chemotherapy (v) Patients not willing to provide the consent for study (vi) Patients who did not complete the course of treatment

**Table 2:** Categorization of the questions.

S. no.	Symptoms	EORTC (question number)	MFIQ (question number)
1.	Chewing problems	15	4, 5, 6, 10, 12, 13, 14, 15
2.	Trismus	9	3, 11
3.	Dysphagia	5, 6, 7	8
4.	Pain	1, 2, 3, 4, 21	Nil
5.	Sensory impairment	11, 12	Nil
6.	Xerostomia	10	Nil
7.	Social activities	16, 18, 19, 20	1, 2, 7, 9
8.	Psychological score	8, 13, 14, 17	Nil

**Table 3:** Scoring and grading of oral symptoms.

Symptoms	Mild	Moderate	Severe
1. Chewing disabilities	9 ≤ 18	19 ≤ 27	28 ≤ 36
2. Trismus	3 ≤ 6	7 ≤ 9	9 ≤ 12
3. Dysphagia	4 ≤ 8	9 ≤ 12	13 ≤ 16
4. Pain	5 ≤ 10	11 ≤ 15	16 ≤ 20
5. Sensory difficulties	2 ≤ 4	5 ≤ 6	7 ≤ 8
6. Sticky saliva	1	2 ≤ 3	3 ≤ 4
7. Social activities	8 ≤ 16	17 ≤ 24	25 ≤ 3 2
8. Psychological burden	4 ≤ 8	9 ≤ 12	13 ≤ 16

**Table 4:** Summary of the clinical and treatment details of participants.

Category		*n* (%)
Tumor localization	Buccal mucosa Tongue Oropharynx Floor of the mouth Retromolar trigone Soft palate Maxilla Mandible	10 (10.3%) 34 (35%) 12 (12.4%) 18 (18.6%) 3 (3.1%) 2 (2.1%) 12 (12.4%) 6 (6.2%)

Staging	Stage 1 Stage 2 Stage 3 Stage 4	15 (16%) 33(34%) 35(36%) 14(14%)

Mode of treatment	Surgery alone Surgery + radiotherapy Surgery + radiotherapy + chemotherapy	3 (3%) 64 (66%) 30 (31%)

**Table 5:** Association between various symptoms and malnutrition.

Symptoms	Prevalence	Percentage in which malnutrition is absent	Percentage in which malnutrition is present	*p* value
Chewing problem	32	56.25	43.75	0.617
Trismus	37	62.16	37.84	0.709
Dysphagia	74	56.76	43.24	0.274
Pain	79	59.49	40.50	0.899
Taste disturbance	37	46	54	**0.029**
Xerostomia	91	61.53	38.47	0.172
Social activity	72	56.94	43.06	0.331
Psychological	69	62.32	37.68	0.426

**Table 6:** Association between the oral symptoms, psychological burden, and impaired social activities with various modes of treatment.

Symptoms	Surgery + CT + RT (%)	Surgery + RT (%)	Surgery alone (%)	*p* value
Chewing problems	16.70	39.10	66.70	**0.045**
Trismus	16.70	48.40	33.30	**0.012**
Dysphagia	60.00	82.80	100.00	**0.033**
Pain	93.30	76.60	66.70	0.12
Taste disturbance	33.30	40.60	33.30	0.782
Sticky saliva	80.00	100.00	100.00	**0.001**
Social activity	80.00	70.30	100.00	0.354
Psychological	83.30	65.60	66.70	0.207

## Data Availability

Supporting data are available from the corresponding author on request.

## Abbreviations

QOL: Quality of life
MFIQ: Mandibular Function Impairment Questionnaire
EORTC QOL-H&N35: European Organization for the Research and Treatment of Cancer's Quality of Life Questionnaire and the Head and Neck35.

## References

[1] Nagy J., Braunitzer G., Antal M., Berkovits C., Novák P., Nagy K. (2014). Quality of life in head and neck cancer patients after tumor therapy and subsequent rehabilitation: an exploratory study. *Quality of Life Research*.

[2] Ferlay J., Ervik M., Lam F. (2018). *Global Cancer Observatory*.

[3] Cognetti D. M., Weber R. S., Lai S. Y. (2008). Head and neck cancer: an evolving treatment paradigm. *Cancer*.

[4] Pulte D., Brenner H. (2010). Changes in survival in head and neck cancers in the late 20th and early 21st century: a period analysis. *The Oncologist*.

[5] Mehanna H., West C. M. L., Nutting C., Paleri V. (2011). Head and neck cancer-part 2: treatment and prognostic factors. *Clinical Otolaryngology*.

[6] Thapa R., Wilson G. D. (2016). Head and neck cancer: current treatment options and associated challenges. *Scholars Journal of Applied Medical Sciences*.

[7] Nguyen N. P., Sallah S., Karlsson U., Antoine J. E. (2002). Combined chemotherapy and radiation therapy for head and neck malignancies: quality of life issues. *Cancer*.

[8] Braakhuis B. J. M., Brakenhoff R. H., Rene Leemans C. (2012). Treatment choice for locally advanced head and neck cancers on the basis of risk factors: biological risk factors. *Annals of Oncology*.

[9] Kamstra J. I., Jager-Wittenaar H., Dijkstra P. U. (2011). Oral symptoms and functional outcome related to oral and oropharyngeal cancer. *Support Care Cancer*.

[10] Gritz E. R., Carmack C. L., de Moor C. (1999). First year after head and neck cancer: quality of life. *Journal of Clinical Oncology*.

[11] KA- Hassanein AM., Musgrove BT., Bradbury E. (2005). Psychological outcome of patients following treatment of oral cancer and its relation with functional status and coping mechanisms. *Journal of Cranio-Maxillofacial Surgery*.

[12] Rinkel R. N., Verdonck-de Leeuw I. M., Langendijk J. A., van Reij E. J., Aaronson N. K., René Leemans C. (2009). The psychometric and clinical validity of the SWAL-QOL questionnaire in evaluating swallowing problems experienced by patients with oral and oropharyngeal cancer. *Oral Oncology*.

[13] Nazar G., Garmendia M. L., Royer M., McDowell J. A., Weymuller E. A., Yueh B. (2010). Spanish validation of the University of Washington quality of life questionnaire for head and neck cancer patients. *Otolaryngology-Head and Neck Surgery*.

[14] Scharloo M., de Jong R. J. B., Langeveld T. P. M., Van Velzen-Verkaik E., den Akker M. M. D.-O., Kaptein A. A. (2010). Illness cognitions in head and neck squamous cell carcinoma: predicting quality of life outcome. *Supportive Care in Cancer*.

[15] Thomas L., Jones T. M., Tandon S., Tandon P., Lowe D., Rogers S. (2009). Speech and voice outcomes in oropharyngeal cancer and evaluation of the University of Washington quality of life speech domain. *Clinical Otolaryngology*.

[16] Vartanian J. G., Carvalho A. L., Yueh B. (2004). Long-term quality-of-life evaluation after head and neck cancer treatment in a developing country. *Archives of Otolaryngology Head & Neck Surgery*.

[17] Rathod S., Gupta T., Ghosh-Laskar S., Murthy V., Budrukkar A., Agarwal J. (2013). Quality-of-life (QOL) outcomes in patients with head and neck squamous cell carcinoma (HNSCC) treated with intensity-modulated radiation therapy (IMRT) compared to three-dimensional conformal radiotherapy (3D-CRT): evidence from a prospective randomized study. *Oral Oncology*.

[18] De Graeff A., De Leeuw J. R., Ros W. J., Hordijk G. J., Blijham G. H., Winnubst J. A. (2000). Pretreatment factors predicting quality of life after treatment for head and neck cancer. *Head & Neck*.

[19] Bjordal K., Hammerlid E., Ahlner-Elmqvist M. (1999). Quality of life in head and neck cancer patients: validation of the European organization for research and treatment of cancer quality of life questionnaire-H&N35. *Journal of Clinical Oncology*.

[20] Rettig E. M., D’Souza G., Thompson C. B., Koch W. M., Eisele D. W., Fakhry C. (2016). Health-related quality of life before and after head and neck squamous cell carcinoma: analysis of the surveillance, epidemiology, and end results-medicare health outcomes survey linkage. *Cancer*.

[21] Hammerlid E., Bjordal K., Ahlner-Elmqvist M. (2001). A prospective study of quality of life in head and neck cancer patients. Part I: at diagnosis. *The Laryngoscope*.

[22] Terrell J. E., Welsh D. E., Bradford C. R. (2000). Pain, quality of life, and spinal accessory nerve status after neck dissection. *The Laryngoscope*.

[23] Trotti A., Bellm L. A., Epstein J. B. (2003). Mucositis incidence, severity and associated outcomes in patients with head and neck cancer receiving radiotherapy with or without chemotherapy: a systematic literature review. *Radiotherapy and Oncology*.

[24] Biazevic M. G. H., Antunes J. L. F., Togni J., de Andrade F. P., de Carvalho M. B., Wünsch-Filho V. (2008). Immediate impact of primary surgery on health-related quality of life of hospitalized patients with oral and oropharyngeal cancer. *Journal of Oral and Maxillofacial Surgery*.

[25] Shepherd K. L., Fisher S. E. (2004). Prospective evaluation of quality of life in patients with oral and oropharyngeal cancer: from diagnosis to three months post-treatment. *Oral Oncology*.

[26] Agarwal S. K., Munjal M., Koul R., Agarwal R. (2014). Prospective evaluation of the quality of life of oral tongue cancer patients before and after the treatment. *Annals of Palliative Medicine*.

[27] McLaughlin L. (2013). Taste dysfunction in head and neck cancer survivors. *Oncology Nursing Forum*.

[28] Ogama N., Suzuki S., Umeshita K. (2010). Appetite and adverse effects associated with radiation therapy in patients with head and neck cancer. *European Journal of Oncology Nursing*.

[29] Ogama N., Suzuki S. (2012). Adverse effects and appetite suppression associated with particle beam therapy in patients with head and neck cancer. *Japan Journal of Nursing Science*.

[30] Jager-Wittenaar H., Dijkstra P. U., Vissink A., van Oort R. P., van der Laan B. F. A. M., Roodenburg J. L. N. (2011). Malnutrition in patients treated for oral or oropharyngeal cancer-prevalence and relationship with oral symptoms: an explorative study. *Supportive Care in Cancer*.

[31] Gellrich N.-C., Schramm A., Böckmann R., Kugler J. (2002). Follow-up in patients with oral cancer. *Journal of Oral and Maxillofacial Surgery*.

[32] Kubrak C., Olson K., Jha N. (2010). Nutrition impact symptoms: key determinants of reduced dietary intake, weight loss, and reduced functional capacity of patients with head and neck cancer before treatment. *Head Neck*.

[33] Ravasco P., Monteiro-Grillo I., Marques Vidal P., Camilo M. E. (2005). Impact of nutrition on outcome: a prospective randomized controlled trial in patients with head and neck cancer undergoing radiotherapy. *Head & Neck*.

[34] van den Berg M. G. A., Rasmussen-Conrad E. L., van Nispen L., van Binsbergen J. J., Merkx M. A. W. (2008). A prospective study on malnutrition and quality of life in patients with head and neck cancer. *Oral Oncology*.

